# Single-Nucleotide Polymorphisms in Thrombotic Thrombocytopenic Purpura: A Genetic Predisposition to Immune Thrombotic Thrombocytopenic Purpura

**DOI:** 10.7759/cureus.75202

**Published:** 2024-12-06

**Authors:** Alexander Konopnicki, Ariel Rischall, Bagi Jana

**Affiliations:** 1 Internal Medicine, University of Texas Medical Branch, Galveston, USA; 2 Hematology and Medical Oncology, Saint Louis University School of Medicine, St. Louis, USA; 3 Hematology and Oncology, University of Texas MD Anderson Cancer Center, Galveston, USA

**Keywords:** adamts 13, hereditary ttp, immune ttp, next generation sequencing (ngs), single nucleotide polymorphism

## Abstract

There are two main classifications for thrombotic thrombocytopenic purpura (TTP): immune and hereditary. The majority of TTP cases are immune in nature and are due to inhibitor autoantibodies against ADAMTS13. Hereditary TTP is caused by biallelic pathogenic variants in the ADAMTS13 gene. Immune TTP is treated with therapeutic plasma exchange that both removes inhibitor autoantibodies and repletes deficient ADAMTS13. In hereditary TTP, the treatment is plasma infusion, as there are no inhibitor autoantibodies to remove. Our case report presents a 72-year-old male who experienced recurrent episodes of TTP and whose first episode was complicated by an occipital lobe stroke. Next-generation sequencing revealed three separate homozygous single-nucleotide polymorphisms (SNPs) in exon 1, exon 12, and exon 16 of the ADAMTS13 gene. Inhibitor autoantibody testing was negative in recurrent episodes, and treatment with plasma infusions for hereditary TTP led to relapses. The objective of our case report is to explore the combined effects of these SNPs on ADAMTS13 activity and antigen levels and question if these genetic variations can lower the threshold for an immune TTP episode. Future research is required to determine if lower-than-normal baseline ADAMTS13 activity levels can predispose a patient to TTP and the long-term complications associated with it.

## Introduction

Thrombotic thrombocytopenic purpura (TTP) is caused by severely deficient activity of the ADAMTS13 protease, defined as an activity level of less than 10% [1]. Most cases of TTP are immune in nature and are due to inhibitor autoantibodies against ADAMTS13. This accounts for approximately 95% of cases [2]. Hereditary TTP is much less common than immune TTP and is caused by biallelic pathogenic variants in the ADAMTS13 gene [3]. Due to the high probability of inhibitor presence, the first-line treatment for a presumed case of TTP is therapeutic plasma exchange (TPE). TPE works by both replacing the deficient ADAMTS13 and removing inhibitor autoantibodies against ADAMTS13 [4]. For patients with high confidence in the diagnosis of hereditary TTP, the recommendation is plasma transfusion rather than TPE [5].

The human ADAMTS13 gene contains 29 exons on chromosome 9q34 [6]. Levy et al. were the first to demonstrate that deficiency of ADAMTS13 is the molecular mechanism responsible for TTP. They identified several single-nucleotide polymorphisms (SNPs) associated with amino acid substitutions [6]. The identified SNPs included the mutations found in our patient: R7W, Q448E, and P618A.

Analysis of the individual effects of these amino acid alterations showed that several sequence variations can interact with each other, altering the phenotype of ADAMTS13 deficiency. R7W and Q448E have been shown to have minimal effects on the secretion of ADAMTS13. However, P618A has been shown to greatly reduce ADAMTS13 activity and antigen levels. R7W and Q448E were found to be positive modifiers of ADAMTS13 secretion in the context of P618A but were unable to rescue the severely reduced activity conferred by P618A. The mean antigen level in vitro with the combination of these three SNPs was found to be 63% ± 13% of wild-type ADAMTS13, while the mean ADAMTS13 activity level was 32% ± 2% of wild-type ADAMTS13 [7].

## Case presentation

A 72-year-old male with a past medical history significant for intermittent thrombocytopenia, transient ischemic attack (TIA), chronic kidney disease (CKD), and TTP complicated by right occipital lobe stroke presented to the oncology clinic for a three-month follow-up for his history of recurrent TTP. His family history was significant for a son who was diagnosed with immune TTP with positive inhibitor autoantibody testing.

The patient originally presented with new-onset vision changes two years prior. At that time, imaging and workup revealed a right occipital lobe stroke with right posterior cerebral artery occlusion. His labs were significant for a platelet count less than 10,000/μL (reference range: 150,000-450,000/μL), indirect bilirubin of 2.2 mg/dL (reference range: 0.1-1.1 mg/dL), creatinine of 1.6 mg/dL (reference range: 0.6-1.25 mg/dL), international Normalized Ratio (INR) of 1.1 (reference range: <1.1), and mean corpuscular volume (MCV) of 88.9 fL (reference range: 81.7-95.6 fL). His peripheral smear showed evidence of microangiopathic hemolytic anemia (MAHA) with 1+ schistocytes. His PLASMIC score for TTP on admission was 7, which designated a high risk of severe ADAMTS13 deficiency. His ADAMTS13 activity was ultimately found to be less than 5% (reference range: >61%).

He was transferred to our hospital for initiation of TPE after experiencing seizure-like activity that required intubation despite receiving plasma infusions at the outside hospital. He received three sessions of TPE and glucocorticoid therapy with a subsequent platelet increase to more than 150,000/μL (reference range: 150,000-450,000/μL). Of note, his inhibitor screen was negative, which pointed to the possibility of hereditary TTP.

He was readmitted to the hospital one month later with recurrent thrombocytopenia, concerning relapsed TTP. His ADAMTS13 activity was found to be 57% (reference range: >61%). He was treated with plasma infusions with significant improvement in his platelets to greater than 200,000/μL (reference range: 150,000-450,000/μL). He was discharged with plans for weekly plasma transfusions as a treatment for hereditary TTP.

**Figure 1:**
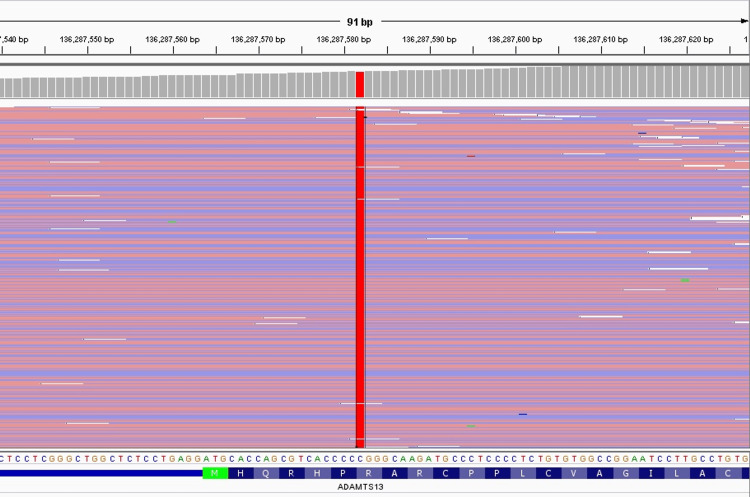
NGS analysis depicting the P618A SNP in exon 16 of ADAMTS13 (performing laboratory: Versiti Diagnostic Laboratories, Hematology Genetics) NGS: next-generation sequencing; SNP: single nucleotide polymorphism

**Figure 2:**
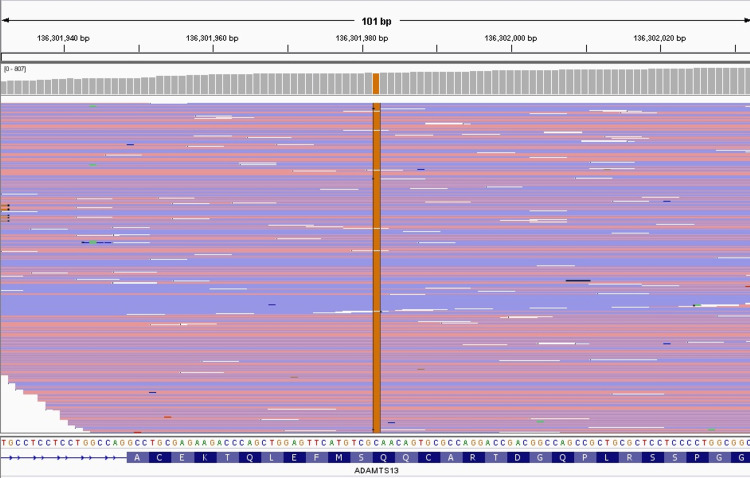
NGS analysis depicting the Q448E SNP in exon 12 of ADAMTS13 (performing laboratory: Versiti Diagnostic Laboratories, Hematology Genetics) NGS: next-generation sequencing; SNP: single nucleotide polymorphism

**Figure 3:**
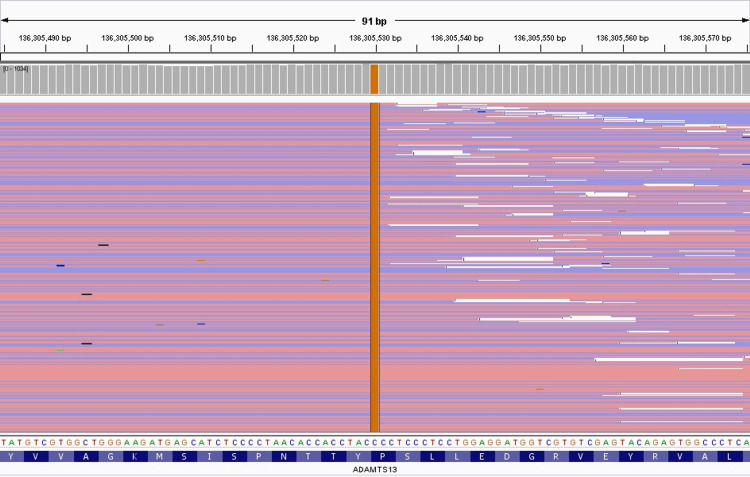
NGS analysis depicting the R7W SNP in exon 1 of ADAMTS13 (performing laboratory: Versiti Diagnostic Laboratories, Hematology Genetics) NGS: next-generation sequencing; SNP: single nucleotide polymorphism

Next-generation sequencing (NGS) of ADAMTS13 was obtained, which demonstrated likely pathogenic homozygous mutations in exon 1 (R7W), exon 12 (Q448E), and exon 16 (P618A) (Table 1). The NGS analysis of each SNP is illustrated in Figures 1-3.

**Table 1:** ADAMTS13 sequencing results demonstrating homozygous, likely pathogenic mutations in exon 1 (R7W), exon 12 (Q448E), and exon 16 (P618A)

Gene transcript	Exon	DNA change	Amino acid change	Zygosity	Pathogenicity	Genomic coordination
ADAMTS13 NM_139025.4	Exon 1	c.19C>T	p.Arg7Trp (R7W)	Homozygous	Likely pathogenic	Chr9 g.136287582C>T
ADAMTS13 NM_139025.4	Exon 12	c.1342C>G	p.Gln448Glu (Q448E)	Homozygous	Likely pathogenic	Chr9 g.136301982C>G
ADAMTS13 NM_139025.4	Exon 16	c. 1852C>G	p.Pro618Ala (P618A)	Homozygous	Likely pathogenic	Chr9 g.136305530C>G

Several weeks later, the patient developed recurrent thrombocytopenia and was readmitted to the hospital. His repeat inhibitor testing was negative during this admission, and his ADAMTS13 level was again low at 29% (reference range: >61%). Given the patient’s lack of response to plasma transfusions, it was suspected the patient may have an inhibitor that could not be detected on available testing. He was treated with a glucocorticoid taper and received three TPE sessions. His thrombocytopenia resolved, and the plasma infusions were stopped upon discharge. The patient completed his steroid taper and received six cycles of weekly rituximab thereafter.

Since discharge, the patient’s platelet counts have remained stable within the normal range. His most recent ADAMTS13 activity was slightly decreased at 42% (reference range: >61%), likely representing his baseline.

## Discussion

Our patient’s most recent ADAMTS13 activity level was measured to be low at 42%, likely representing his baseline. A normal ADAMTS13 activity for a healthy adult is between 50% and 160% [8]. Based on our patient’s genetic mutations, it is likely his constellation of SNPs accounts for his lower-than-baseline ADAMTS13 activity level.

Sonneveld et al. illustrated the relationship between low ADAMTS13 activity and the risk of ischemic stroke [9]. Our patient’s medical history was significant for a TIA and intermittent thrombocytopenia prior to his first episode of TTP. While our patient’s ADAMTS13 mutation does not constitute true hereditary TTP, he may be predisposed to similar complications based on his underlying ADAMTS13 deficiencies.

Despite our patient’s lower-than-normal baseline ADAMTS13 activity level, his ADAMTS13 activity level was less than 10% on initial presentation, indicating his initial episode of TTP was immune in nature. His ADAMTS13 SNPs have been shown to lead to moderately reduced ADAMTS13 activity levels, but not enough to constitute true TTP. When his diagnosis was presumed to be hereditary TTP and he was treated with weekly plasma infusion therapy, his disease relapsed, which led to an additional hospital admission. This points to an underlying impetus triggering an episode of TTP in a patient with baseline decreased ADAMTS13 activity.

The homozygous nature of his mutations may also account for his son’s development of TTP. Our patient’s son differed from his father in that his inhibitor autoantibody testing was positive on initial presentation. This both suggests the presence of an undetected inhibitor autoantibody present in our patient and makes one question whether this population is more prone to the development of antibodies against ADAMTS13.

The differentiating factor between hereditary and immune TTP in guiding the treatment of subsequent episodes is the identification of inhibitor autoantibodies in immune TTP. Patients with ADAMTS13 deficiency are likely to have dysfunctional ADAMTS13 with alterations of either enzymatic structure or activity leading to negative inhibitor autoantibody testing [10]. Our patient’s homozygous pathogenic SNPs could lessen the likelihood of inhibitor antibody identification.

The timing of inhibitor testing is also paramount because both plasma infusion and TPE can remove or dilute inhibitors in the patient’s plasma [11]. Our patient received plasma infusion therapy at the outside hospital prior to his transfer. This was the only time our patient had an ADAMTS13 activity level of less than 10%. The inhibitor autoantibodies may have been diluted by his pre-hospital treatment.

Another consideration is the utility of routine testing for ADAMTS13 antigen level to assess the patient’s disease status. Deficiency of ADAMTS13 activity has been proven to be beneficial for the diagnosis of idiopathic TTP at the time of acute disease. However, Yang et al. demonstrated a severe depletion of ADAMTS13 antigen level during acute disease was associated with mortality. In patients who achieved initial clinical responses, ADAMTS13 antigen levels appeared to be restored faster than ADAMTS13 activity. Further analysis demonstrated that the ADAMTS13 antigen level at the time of initial clinical recovery was significantly higher in patients who achieved sustained clinical remission than in the group who soon after had an exacerbation [12]. Patients recovering from an acute episode of TTP are presumed to recover their ADAMTS13 activity and antigen level back to the normal level of 60-150%. Our patient had both decreased ADAMTS13 activity and presumed antigen based on the previous studies of his mutations. This patient population may be predisposed to relapse after the initial event due to both the rate of recovery to a lower baseline ADAMTS13 antigen and activity level.

## Conclusions

Our case report presents a unique population of patients who have homozygous ADAMTS13 SNPs causing moderate deficiencies in both ADAMTS13 activity and antigen level. There needs to be an increased awareness that the identification of ADAMTS13 mutations does not inherently constitute hereditary TTP when deciding on treatment plans. Additional research needs to be aimed at this population to investigate if the threshold to trigger an episode of immune TTP is lowered, as well as a propensity for relapse and recurrent episodes in the setting of decreased baseline ADAMTS13 activity and antigen levels. It also brings attention to the potential utility of routine testing for ADAMTS13 antigen level, in addition to ADAMTS13 activity level and inhibitor autoantibody testing.

## References

[1] George JN, Nester CM (2014). Syndromes of thrombotic microangiopathy. N Engl J Med.

[2] Blombery P, Scully M (2014). Management of thrombotic thrombocytopenic purpura: current perspectives. J Blood Med.

[3] Kremer Hovinga JA, George JN (2019). Hereditary thrombotic thrombocytopenic purpura. N Engl J Med.

[4] Zheng XL, Vesely SK, Cataland SR (2020). ISTH guidelines for treatment of thrombotic thrombocytopenic purpura. J Thromb Haemost.

[5] Page EE, Kremer Hovinga JA, Terrell DR, Vesely SK, George JN (2017). Thrombotic thrombocytopenic purpura: diagnostic criteria, clinical features, and long-term outcomes from 1995 through 2015. Blood Adv.

[6] Levy GG, Nichols WC, Lian EC (2001). Mutations in a member of the ADAMTS gene family cause thrombotic thrombocytopenic purpura. Nature.

[7] Plaimauer B, Fuhrmann J, Mohr G (2006). Modulation of ADAMTS13 secretion and specific activity by a combination of common amino acid polymorphisms and a missense mutation. Blood.

[8] Tso AC, Sum CL, Ong KH (2022). Reference range for ADAMTS13 antigen, activity and anti-ADAMTS13 antibody in the healthy adult Singapore population. Singapore Med J.

[9] Sonneveld MA, de Maat MP, Portegies ML (2015). Low ADAMTS13 activity is associated with an increased risk of ischemic stroke. Blood.

[10] Levy GG, Motto DG, Ginsburg D (2005). ADAMTS13 turns 3. Blood.

[11] Tsai HM, Raoufi M, Zhou W (2006). ADAMTS13-binding IgG are present in patients with thrombotic thrombocytopenic purpura. Thromb Haemost.

[12] Yang S, Jin M, Lin S, Cataland S, Wu H (2011). ADAMTS13 activity and antigen during therapy and follow-up of patients with idiopathic thrombotic thrombocytopenic purpura: correlation with clinical outcome. Haematologica.

